# Determinants of delayed detection of cancers in Texas Counties in the United States of America

**DOI:** 10.1186/1475-9276-11-29

**Published:** 2012-05-29

**Authors:** Gordon Gong, Eric Belasco, Kristopher A Hargrave, Conrad P Lyford, Billy U Philips

**Affiliations:** 1 F. Marie Hall Institute for Rural Community Health, Texas Tech University Health Science Center, Lubbock, TX 79430, USA; 2 Department of Agricultural Economics and Economics, Montana State University, Bozeman, MT 59717-2920, USA; 3 Department of Agricultural and Applied Economics, Texas Tech University, Lubbock, TX 79409, USA; 4 Department of Family and Community Medicine, F. Marie Hall Institute for Rural and Community Health, Texas Tech University Health Science Center, Suite 2B440 - 3601 4th Street MS 6232, Lubbock, TX 79430-6232, USA

**Keywords:** Early detection, Cancer, Socioeconomic, Deprivation, Wellbeing index

## Abstract

**Introduction:**

Previous studies have shown that delayed detection of several cancers is related to socioeconomic deprivation as measured by the Wellbeing Index (WI) in Texas, the United States of America (USA). The current study investigates whether delayed cancer detection is related to lack of health insurance, physician shortage and higher percentages of Hispanics rather than WI *per se* since these factors are directly related to delayed cancer detection and may confound WI.

**Methods:**

Cancer data and potential determinants of delayed cancer detection are derived from Texas Cancer Registry, Texas State Data Center, and Texas Department of State Health Services and U.S. Census Bureau. Texas cancer data from 1997 to 2003 are aggregated to calculate age-adjusted late- and early-stage cancer detection rates. The WI for each county is computed using data from the USA Census 2000. A weighted Tobit regression model is used to account for population size and censoring. The percentage of late-stage cancer cases is the dependent variable while independent variables include WI and the aforementioned potential confounders.

**Results:**

Delayed detection of breast, lung, colorectal and female genital cancers is associated with higher percentage of uninsured residents (p < 0.05). Delayed detection is also associated with physician shortage and lower percentages of Hispanics for certain cancers *ceteris paribus* ( *p* < 0.05). The percentage of late-stage cases is positively correlated with WI for lung, and prostate cancers after adjusting for confounders ( *p* < 0.05).

**Conclusions:**

The percentages of uninsured and Hispanic residents as well as physician supply are determinants of delayed detection for several cancers independently of WI, and *vice versa*. Identification of these determinants provides the evidence-base critical for decision makers to address specific issues for promoting early detection in effective cancer control.

## Introduction

Identifying cancer in its relatively early stage is critical for cancer control and leads to substantially higher success rates in treatment and fewer cancer deaths [1,2]. In the past two decades, cancer mortality rates have declined significantly mainly due to reduction in the number of smokers, increased cancer screening, and better treatment [2]. However, despite the impact of cancer education programs [3] including recent interactive computer-based education [4] for early detection and treatment, large gaps exist in cancer awareness, early detection and treatment among different ethnic groups and social classes [2,5]. Barriers to early cancer detection need to be identified and overcome for effective cancer control.

Health status and health disparities among different social and ethnic groups are to a large degree determined by socioeconomic status in general [6,7]. Socioeconomic factors are particularly important determinants for cancer mortality [2]. For example, health behavior including regular checkups and participation in cancer surveillance among high risk groups may be determined by health insurance coverage which is often absent in socioeconomically deprived individuals. To quantify socioeconomic deprivation for a given community (e.g., census track), Albrecht and Ramasubramanian developed a Wellbeing Index (WI) by principal component analysis using ten socioeconomic variables from United States of America (USA) Census 2000 data [8]. The WI was subsequently shown to be highly correlated with delayed cancer detection (as assessed by the ratio of late- to early-stage cases) of female genital system, lung-bronchial and all-type cancers at diagnosis among Texas counties in USA [9]. However, it is possible that this correlation may actually reflect a potential correlation between delayed cancer detection and lack of insurance and/or shortage of physicians, etc. In other words, these factors may confound socioeconomic deprivation and play a role in delayed cancer detection independent of socioeconomic status or in concert with it. For example, Roetzheim et al. have found that uninsured cancer patients in Florida of United States were more likely diagnosed at a late-stage for colorectal, breast and prostate cancers, while patients with Medicaid were more likely diagnosed at a late stage of breast cancer and melanoma [10]. In addition, African-Americans were more likely diagnosed with late stage breast and prostate cancers than non-Hispanic whites [10]. At the national level, Halpern et al. have reported that advanced stage at diagnosis of 12 cancers is more often observed among uninsured or Medicaid-insured patients in the United States as compared with patients with private insurance. Also, higher percentages of black and Hispanic patients tend to have late-stage cancers regardless of insurance status [11]. Because 16.3% (or 49.9 million) of the population in the United State had no health insurance in 2010, the impact of lack of insurance on cancer control is very large [12]. Additionally, higher ratio of physicians to population served is associated with lower incidence of late-stage colorectal cancer [13]. Identification of specific factors associated with cancer surveillance and cancer control behaviors (such as screening mammography and ultrasound examination, colonoscopy, regular timely medical checkup, etc.) and socioeconomic inequality is critical for reducing cancer deaths and health disparities [2] because early cancer detection is the key to cancer control. For example, American Cancer Society data show that five-year survival rate decreases from 88% at stage I to 15% at stage IV for breast cancer, from 76% at stage I to 6% at stage IV for colon cancer, and from 49% at stage to 1% at stage IV for lung cancer [14]. To promote early cancer detection, the National Cancer Institute (NCI) has initiated the Early Detection Research Network (EDRN) with dozens of participating institutions aiming at developing new ways of testing cancer in its earliest stages for cancer control [15]. The study reported here is undertaken to determine whether socioeconomic deprivation measured by WI is an determinant of delayed diagnosis of lung-bronchial, breast, prostate, female genital system, and colorectal cancers independently of ethnicity, health insurance coverage, and physician supply, and *vice versa*. These latter factors may be more closely and directly related to cancer surveillance activities at the community (such as county) level than WI *per se*. Identification of determinants of delayed cancer detection associated with socioeconomic inequality will provide the evidence-base critical for decision makers to establish policies to promote early detection for effective cancer control.

## Methods

### Data sources

This study was approved by Texas Tech University Health Sciences Center Institutional Review Board with exemption for review because of its use of published data. Ten socioeconomic variables for constructing the WI are derived from the USA census 2000 with details reported previously [8-10]. We use the Census 2000 rather than 2010 data for the purpose of comparison with previous results that used Census 2000 data [16] and to make the data more historically contiguous with the dependent variable. These ten socioeconomic variables include public income support, disabilities, homeownership, bedroom overcrowding, educational attainment, single parental household, poverty status, vehicle ownership, unemployment, and home telephone service [8,9,16]. Data for cancer stage at diagnosis are provided by the Texas Cancer Registry, Cancer Epidemiology and Surveillance Branch, Texas Department of State Health Services, USA [17]. This database provides data by year, age, Hispanic origin, etc. as well as population size for each county from which to calculate age-adjusted early- and late-stage cancer prevalence and percentage of Hispanics. We use data for lung-bronchial, breast, prostate, female genital system, and colorectal cancers from 2000 as well as data three years before and three years after 2000 (i.e., data from 1997 to 2003) in order to be consistent with socioeconomic data derived from USA census 2000 assuming that these socioeconomic variables do not deviate from 2000 census data significantly 3 year before and after 2000.

Data for the percentages of the uninsured are obtained from Texas State Data Center and U.S. Census Bureau [18,19]. The number of physicians and estimated population size in each county from 1997 to 2003 are derived from Texas Department of State Human Services (DSHS) [20]. Physician supply is the number of physicians per 1,000 residents in each county. Physicians considered are those with medical doctor (MD) and/or doctor of osteopathy (DO) degrees who worked directly with patients. Residents and fellows; teachers; administrators; researchers; and those who were working for the federal government, military, retired, or not in practice were excluded from the total of physicians by DSHS [20]

### Statistical analysis

These ten socioeconomic variables (see Additional file 1: Table S5) from USA Census 2000 are subjected to a principal component analysis (PCA) with each of the 254 Texas counties as a unit of observation following the PRINCOMP procedure of the SAS statistical package (Cary, NC). The first principal component scores of the 254 counties are used as a continuous variable for WI (rather than grouping the scores according to deciles in an ordinal scale from 1 to 10 as done in [8]) with larger values indicating a higher degree of socioeconomic deprivation. The first principle component scores are standardized with mean of 0, and one standard deviation as 1, ranging from −2.3 to 4.5 in the present study. The first principle component accounts for 51% of total variance in the present study. PCA is used when variables are highly correlated as is the case in the present study, which reduces the observed variables to a smaller number of principal components. In this study, we use only the first principle component as a composite index to represent the 10 socioeconomic variables.

We pool the seven-year (1997–2003) data to calculate the numbers of age-adjusted late- and early-stage cancer cases per unit (100,000) population using 2000 USA standard population [21]. Pooling the 7-year data reduces the dramatic variability in rates seen in any single year especially in counties with a smaller population. We use the percentage of late-stage cases among all staged cancer cases in our analysis. The number of unstaged cancer cases is not included in the denominator because the percentage of unstaged cases varies significantly by cancer type as well as by county, and inclusion of such cases in the denominator would result in uncertainty in estimating the percentage of late-stage cancer cases. Carcinoma *in situ* and localized cancers are considered as early-stage while cancers defined as “regional, direct extension only”, “regional, regional lymph nodes only”, “regional, direct extension and regional lymph nodes”, “regional, NOS” and “distant” are considered as late-stage [22,23].

After data from seven years are pooled for each cancer type, there still exist a number of counties that have no single late-stage and/or early-stage case for certain cancers because of small population sizes. Therefore, a doubly-bounded censoring mechanism is introduced whereby the percentages of late-stage cancer cases at 0% (only early-stage cancer cases occur) or 100% (only late-stage cancer cases occur) are censored. Since this percentage is censored, the dependent variable is not fully observed (or latent) at all possible values of independent variables. As a result, typical least squares methods would be inadequate and may lead to biased estimation [24]. To account for this censoring, the Tobit regression model (with the SAS QLIM procedure) is used with the logged sum of one plus the percentage of late-stage cancer cases as the response variable and the following as explanatory variables: WI, log of percentage of Hispanics, physician supply, and log of percentage of the uninsured. The purpose of adding one to the percentage of late-stage cancer cases is to avoid the undefined result from taking the natural log of zero. When there is not a single cancer case, the observation is deleted in the analysis since it does not provide any information regarding the percentage of late-stage cancer cases. Deleted observations range from 0 (lung-bronchial cancer) to 6 (female genital system cancer), and censored observations from 0 (lung-bronchial) to 11 (female genital system cancer) out of 254 observations. Since regression residuals for the dependent variable are not normally distributed, a log-transformation is performed to achieve normality before regression analysis.

Weighted statistical methods are used in this study to account for the wide variation in population size among Texas counties. For example, according to the 2000 census, Harris County (where Houston is located) had a population of 3.4 million, while Loving County had a population of 67. Weighted statistics adjust (or weight) the effect of each observation according to population size, so this procedure produces a more representative parameter estimates. If the regression was not weighted (i.e., all county-level observations were treated equally as is the case in typical least squares methods), then the results would be biased toward counties with a smaller population. Also, certain counties may have a single case of early- or late-stage cancer with the percentage of late-stage cases being 0 and 100, respectively, which do not reflect the reality but rather are the results of small population sizes. Such counties are censored in our analysis, and the Tobit model takes account this censoring.

In the Tobit regression model, independent variables include WI, physician supply, log percentage of uninsured and log of percentage of Hispanics. In a second model, log percentage of uninsured is not entered in order to see its influence on the relationship between the percentage of late-stage cases and WI.

## Results

**Figure 1:**
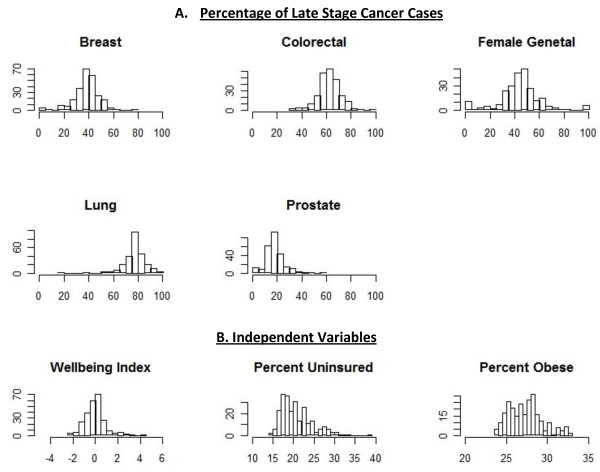
**Empirical Histograms of Relevant Variables, by Texas Counties.A.** X axis is the percentage of late stage cases for each cancer; Y axis is frequency (number of counties). **B.** X axis is the independent variable; Y axis is frequency.

Table 1 shows the weighted summary statistics of variables of interest which is complemented by Figure 1 which shows the empirical histogram associated with all variables of interest. One notable feature of this data from Figure 1 is the wide range in all independent variables.

**Table 1:** Weighted Summary Statistics for Variables of Interest

**Variable**	**Mean**	**Std. dev**	**Min**	**Max**
WI	−0.20	0.14	−2.30	4.49
% Hispanic	31.30	3.34	1.64	97.61
% Uninsured	24.52	1.05	14.20	38.10
Physician Supply per 1,000	1.54	0.10	0.00	3.24
% Late-Stage Cancer Cases				
Breast	38.35	0.36	0.00	76.06
Colorectal	62.14	0.60	34.26	100.00
Female Genital	45.15	0.61	0.00	100.00
Lung - Bronchial	77.20	0.62	18.90	100.00
Prostate	16.25	0.33	0.00	58.10

Std. dev = standard deviation.

Table 2 presents the results of the weighted Tobit model for the percentage of late-stage cancer cases in relation to WI and other explanatory variables. A blank row within each cancer type separates the two sets of parameter estimates in the presence and absence of % uninsured, respectively. We first report the Tobit model results when the percentage of uninsured is entered as an independent variable. The percentages of late-stage cases of lung-bronchial and prostate cancers were significantly higher in counties with higher WI scores, holding other variables constant (*p* < 0.05). Counties with a higher physician supply tended to have lower percentage of late-stage cases of breast, lung-bronchial and prostate cancers ( *p* < 0.05). Counties with higher rates of uninsured individuals tended to have higher rates of breast, colorectal, lung-bronchial and female genital system cancers detected at later stages ( *p* < 0.05). Counties with higher percentage of Hispanics tended to have lower percentage of late-stage cases of colorectal, and lung-bronchial cancers (p < 0.002) *ceteris paribus*.

**Table 2:** Weighted Tobit Results for Log(1 + Percentage of Late-Stage Cancer Cases)

	**Breast**			**Colorectal**		
	**Estimate**	**SE**	***p***	**Estimate**	**SE**	***p***
Intercept	3.190	0.227	<.0001	3.949	0.122	<.0001
WI	0.016	0.012	0.189	0.006	0.007	0.3573
Log %Hispanic	−0.025	0.016	0.1244	−0.027	0.009	0.0018
Physician Supply	−0.432	0.105	<.0001	−0.088	0.057	0.1205
Log %Uninsured	0.205	0.085	0.0158	0.137	0.046	0.0028
Intercept	3.729	0.039	<.0001	4.308	0.021	<.0001
WI	0.037	0.009	<.0001	0.020	0.005	<.0001
Log %Hispanic	0.002	0.012	0.8379	−0.009	0.006	0.1553
Physician Supply	−0.402	0.106	0.0001	−0.068	0.057	0.2345
	Lung-Bronchial			Prostate		
Intercept	3.610	0.079	<.0001	2.626	0.374	<.0001
WI	0.017	0.004	<.0001	0.045	0.020	0.0279
Log %Hispanic	−0.064	0.006	<.0001	−0.025	0.027	0.349
Physician Supply	−0.272	0.037	<.0001	−0.598	0.173	0.0006
Log %Uninsured	0.204	0.030	<.0001	0.127	0.140	0.3646
Intercept	4.147	0.015	<.0001	2.960	0.065	<.0001
WI	0.038	0.003	<.0001	0.058	0.014	<.0001
Log %Hispanic	−0.037	0.004	<.0001	−0.008	0.019	0.6702
Physician Supply	−0.243	0.040	<.0001	−0.580	0.173	0.0008
	Female Genital system					
Intercept	2.642	0.411	<.0001			
WI	−0.031	0.022	0.1636			
Log %Hispanic	−0.011	0.029	0.7216			
Physician Supply	0.212	0.191	0.2684			
Log %Uninsured	0.382	0.154	0.013			
Intercept	3.649	0.072	<.0001			
WI	0.009	0.016	0.5798			
Log %Hispanic	0.041	0.021	0.0573			
Physician Supply	0.268	0.192	0.1636			

The blank row within each cancer type separates the two sets of parameter estimates in the presence and absence of % uninsured, respectively.

Results derived when the percentage of the uninsured is omitted from the Tobit model show that higher percentages of late-stage cases of breast, colorectal, lung-bronchial and prostate cancers are significantly correlated with higher WI values. Notably, the correlation between the two variables for breast and colorectal cancers becomes significant after omitting the percentage of uninsured.

**Figure 2:**
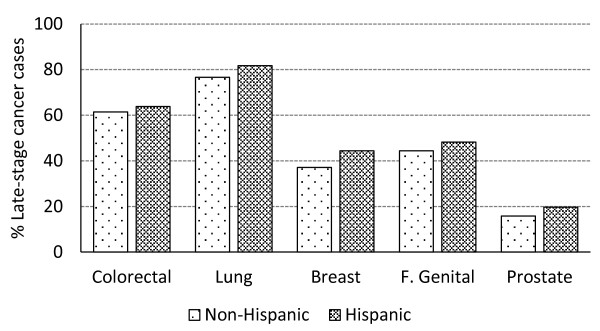
Percentage of late-stage cancer cases in Hispanic vs. non-Hispanics in Texas.

Figure 2 shows that the percentage of late-stage cases of all five categories of cancer except for female genital system cancer is higher in Hispanics than non-Hispanics in Texas as a whole (data from its 254 counties pooled) in the univariate analysis (without adjustment for covariates).

Table 3 presents weighted mean statistics distinguished by % Hispanic population, showing that counties with a higher proportion of Hispanics tend to have a higher degree of socioeconomic deprivation (higher WI), and a higher % uninsured. These independent variables are highly correlated with each other (Table 4).

**Table 3:** Weighted Summary Statistics (Means), grouped by % Hispanics

	**Counties with % Hispanic**		
	**< 15%**	**15% - 50%**	**> 50%**
*n*	112	106	35
Variable	Mean	Mean	Mean
WI	−0.73	−0.40	1.03
% Hispanic	9.15	26.61	70.83
% Uninsured	19.20	25.16	28.95
Physician Supply per 1,000	1.19	1.75	1.35
% Late-Stage Cancer Cases			
Breast	38.51	37.40	40.91
Colorectal	61.94	62.01	62.73
Female Genital	44.24	44.43	48.29
Lung - Bronchial	77.09	76.44	79.53
Prostate	16.23	15.98	17.04

**Table 4:** Pearson’s Correlation Among Independent Variables (R and P Values)

	**WI**	**%Hispanics**	**%Uninsured**	**Physician Sup**
WI	1.0000	0.5910	0.5463	−0.0768
		<.0001	<.0001	0.0836
%Hispanics	0.5910	1.0000	0.8940	−0.1150
	<.0001		<.0001	0.0095
%Uninsured	0.5463	0.8940	1.0000	0.0422
	<.0001	<.0001		0.3431
Physician Sup	−0.0768	−0.1150	0.0422	1.0000
	0.0836	0.0095	0.3431	

This table is a supplement for online-publication only.

## Discussion

Results from this county-level community health study for the first time show that a higher percentage of late-stage cases of lung-bronchial and prostate cancers is correlated with higher degree of socioeconomic deprivation as measured by WI after controlling for several other potential determinants. This finding suggests that socioeconomic deprivation significantly impacts delayed detection of these cancers independently of physician supply and the percentages of the uninsured and Hispanics in Texas counties, USA. Likewise, a higher percentage of late-stage cases of certain cancers is associated with a higher or lower rate of the latter factors independently of socioeconomic deprivation depending on cancer type and covariates (physician supply, percent uninsured or percent Hispanic). It is likely that factors other than those that are studied may be responsible for the correlation between WI and delayed detection of lung-bronchial and prostate cancers such as potentially lower level of education, lack of awareness of cancer control measures, malnutrition, etc. associated with socioeconomic deprivation. Socioeconomic deprivation is such a pervasive determinant of health that it may affect many other factors related to surveillance activities at community (such as county) level. Further studies on such factors are warranted.

Our results also reveal that these independent variables under study may influence delayed cancer detection through different mechanisms along with a certain degree of overlap as reflected in their correlations depending upon cancer type. For example, a higher percentage of late-stage cases of a certain cancer may be correlated with WI because people with a higher degree of socioeconomic deprivation (WI) are more likely to have no health insurance and, therefore, no regular cancer screening for early detection. If health insurance coverage is the main driving force behind the correlation between delayed cancer detection and WI for certain cancers, such as colorectal cancer (CRC), for which screening (e.g., colonoscopy, a very expensive medical procedure) and early detection highly rely on health insurance coverage, then the significant correlation (see Table 2, p < 0.05 for colorectal cancer when% uninsured is not entered into the model) may disappear after adjusting for the percentage of people without health insurance. Health insurance makes a significant difference in early detection of CRC [25]. Similar results are seen for breast cancer as ultrasound and magnetic resonance imaging (MRI) examinations are also expensive. On the other hand, the percentage of late-stage prostate cancer cases is not correlated with the percentage of health insurance coverage in the present study, likely due to the ineffectiveness of the current prostate cancer screening methods (i.e., insurance coverage for prostate cancer screening may not help early detection very much) [9]. The correlation between the percentage of late-stage prostate cancer and WI may be mediated by factors other than health insurance coverage. These findings are important for future policies on achieving health equality in the nation, particularly when coverage of the uninsured has become an important national issue.

Interestingly, previous study showed that WI was significantly associated with delayed detection (assessed by the ratio of late- to early-stage cancer cases similar to the percentage of late-stage cases) of all-type, female genital system, and lung-bronchial cancers but not breast, colorectal and prostate cancers [9], in contrast to the results of the current study. The difference in the results is due to difference in study design with which several covariates of WI are entered in the regression in the current study only. It appears that inclusion of these covariates in the regression model has uncovered certain potential correlations (between WI and delayed detection for breast, lung-bronchial, colorectal and prostate cancers in the absence of percentage of uninsured as a covariate) and revealed that a previous correlation (between WI and delayed detection of female genital system cancer) is likely due to effect of a confounder(s) of WI. This notion is supported by the current finding that WI is highly correlated with other factors studied. A significant correlation between WI and delayed detection for breast and colorectal cancer disappears after inclusion of percentage of the uninsured in the regression model, suggesting the crucial role of health insurance for these two cancers, consistent with findings of Halpern et al. that insurance coverage is particularly important for early detection of these two cancers [11].

The results of the current study also show that Hispanics tended to have higher percentages of late stage cases for all five categories of cancer in Texas in the univariate analysis. This result is consistent with the results of Lantz et al. [26] for breast cancer and with the recent report by American Cancer Society for breast, lung-bronchial, colorectal, and prostate cancers [27]. Lantz et al. further showed that the rate of early detection of breast cancer was still significantly lower in Hispanic women than non-Hispanic white women (this would be equivalent to a higher rate of delayed detection in Hispanic women) after adjustment for socioeconomic factors. However, results from the present study demonstrates that the percentage of late-stage cancers is no longer positively correlated with the percentage of Hispanics after adjustment for socioeconomic deprivation, the percentage of uninsured, and physician supply. The different results from that of Lantz et al. are likely due to the fact that the current study had controlled for more factors such as percentage of the uninsured, and physician supply while studies of Lantz et al. did not. The higher rate in late-stage (or lower rate in early-stage) cancer cases in Hispanics observed with univariate analysis is more likely due to higher degree of socioeconomic deprivation, higher rate of uninsured, etc. since the percentage of late-stage cancer is no longer higher in Hispanics after adjusting for these factors. This result suggests that health disparities related to socioeconomic (such as WI) factors are intertwined with heath disparities among ethnic groups (Hispanics vs. non-Hispanics in our case) (i.e., they are highly correlated in statistical terms).

It should be pointed out that Hispanics are a congregate of people with similar (though not the same) culture, the majority of whom are Mexican Americans in the United States of America, and is not a genetic or racial entity; they may identify themselves as white, black, American Indians, etc. according to U.S. Census Bureau. We did not perform analysis with more ethnic groups for blacks, Native Americans, Asians, etc. because of their relatively small population sizes for most counties. However, we should also point out that Texas is a particularly important state to evaluate the vulnerability and disparity in Hispanics in late-stage cancer detection given its large Hispanic population, which is largely related to socioeconomic deprivation.

This study also demonstrated that higher percentage of late-stage cancer cases is significantly associated with lower physician supply for breast, lung-bronchial, and prostate cancers. This finding emphasizes the importance of physician supplies in early detection of these cancers. Thus, physician supply not only plays an important role in reducing infant mortality [28] but also in early detection of these common cancers. This finding is important to policy makers in their consideration about improving health services in physician shortage areas and underserved communities in Texas. Physician supply is an important component of access to health care in addition to health insurance coverage particularly in a state with severer physician shortage where basic health services are extremely sparse as in many Texas counties.

Recently, the World Health Organization urged member states “to support research on effective policies and interventions to improve health by addressing the social determinants of health.” [29]. The present study provides solid evidence that delay in cancer detection is related to socioeconomic deprivation as well as physician shortage and lack of health insurance. Policy makers should take actions to improve socioeconomic conditions and to reduce disparities in access to health care for early detection of cancers. The socioeconomic variables in this study are readily available from U.S. Census Bureau [16] and other publically available data sources [9,18-20], and can be generally used in community assessments and public health programs including cancer control activities. Similar studies in other states may be performed so that our findings may be generalized beyond Texas.

### Limitations

There are several limitations to consider in our study. First, late vs. early stages are somewhat arbitrarily defined. A cancer defined as late-stage covers a large range of clinical stages, which is not distinguished in the present study. The degree of aggression may be drastically different among different cancer types or among subtypes of the same cancer, which tend to obscure a potential correlation between percentage of late-stage cases and explanatory variables. For example, inflammatory breast cancer or primary squamous cell carcinoma breast cancer may be too aggressive to be detected at early stage even with health insurance coverage and regular check-ups. Secondly, WI is derived from 2000 U.S. Census data, while cancer data have a seven-year span. Although this may be a necessary step to increase population size especially for small counties, socioeconomic status may vary in the seven-year span. However, we drew a WI map based on 2010 U.S. Census data, which is quite similar to the one based on 2000 data (data not shown) in terms of relative rank among counties. Thirdly, the current study uses county as the unit of observation, and population varies drastically from one county to another. Although we used weighted regression, rates (i.e., percent late-stage cancer cases) are subject to a large variation for small counties. Fourthly, most of the independent variables are highly correlated with each other, and the resultant mulitcollinearity tends to inflate variance and *p* values. To reduce the extent of mulitcollinearity, we have eliminated several variables such as rural/urban residence, percentage of obese individuals and Texas regions (east, west and south) from our initial analyses. The final regression model with limited variables reported here is robust in terms of variance inflation factor (<10 as determined by the SAS VIF option, which is acceptable according a commonly accepted rule) [30,31]. Finally, the level of potential environmental exposure to toxins (e.g., pesticides, heavy metals in drinking water) may vary significantly among counties in Texas, which may affect cancer aggressiveness and detection. It would be helpful to perform similar studies in other states to replicate the results reported here.

## Conclusions

Lack of health insurance and physician shortage are important determinants of delayed diagnosis of several cancers independently of socioeconomic deprivation. Socioeconomic deprivation remains associated with delayed diagnosis of several cancers after adjusting for confounders, suggesting its pervasive role as a major barrier to cancer control. Delay in cancer diagnosis in Hispanics is likely due to their higher percentage of the uninsured and higher degree of socioeconomic deprivation because the percentage late-stage cancers is no longer higher in Hispanics after adjusting for WI, rate of uninsured, and physician supply. These determinants of delayed cancer diagnosis provide the evidence-base critical for decision makers to promote early detection and effective cancer control.

## Abbreviations

WI: Wellbeing index; DSHS: Texas Department of State Human Services; NOS: Not otherwise specified; CRC: Colorectal cancer.

## Competing interests

All authors declare no competing interests.

## Authors’ contributions

All the authors contributed to this research. BUP and GG conceived the idea of association between socioeconomic deprivation and delayed cancer detection. EB and GG are responsible for study design and statistical analysis. KAH and CL are responsible for data collection and have participated in development of the manuscript including literature search/review, determinants to be studied, etc. All authors read and approved the final manuscript.

## Authors’ information

GG: Biostatistician;

EB: Biostatistician, Assistant Professor of Economics;

KAH: expert in informatics;

CPL: Associate Professor of Agricultural and Applied Economics;

BUP: Vice President of Texas Tech University Health Sciences Center and Director of F. Marie Hall Institute for Rural & Community Health. Expert in cancer epidemiology, and public health.

## Supplementary Material

Additional file 1 **Table S5.** Variables used to build WI and the percentage of variance explained by each variable in its correlation with the first principal component (%). Click here for file
