# Supplementary material for: Determinants of delayed detection of cancers in Texas Counties in the United States of America
Source: Int J Equity Health. 2012 May 29;11:29. doi: 10.1186/1475-9276-11-29 (PMC3517382; doi:10.1186/1475-9276-11-29)
Supplement: Additional file 1 — Table S5. Variables used to build WI and the percentage of variance explained by each variable in its correlation with the first principal component (%). [file 1475-9276-11-29-S1.doc]

| Table 5. Variables used to build WI and the percentage of variance explained by each variable in its correlation with the first principal component (%) |  |
| --- | --- |
| Variable | % |
| People in households below poverty level | 16.3 |
| People over 18 without High school qualification | 15.1 |
| People in households without car | 13.8 |
| People in households without phone | 11.8 |
| People unemployed | 11.6 |
| People living in homes with too few bed rooms | 9.5 |
| People in single parent households | 7.4 |
| People with any disability | 7.1 |
| People with any form of support | 6.9 |
| People not living in own home | 0.4 |
